# Characterization of *Spirometra erinaceieuropaei* Plerocercoid Cysteine Protease and Potential Application for Serodiagnosis of Sparganosis

**DOI:** 10.1371/journal.pntd.0003807

**Published:** 2015-06-05

**Authors:** Li Na Liu, Zhong Quan Wang, Xi Zhang, Peng Jiang, Xin Qi, Ruo Dan Liu, Zi Fang Zhang, Jing Cui

**Affiliations:** Department of Parasitology, Medical College, Zhengzhou University, Zhengzhou, Henan, People's Republic of China; National Institute of Parasitic Diseases Chinese Center for Diseases Control and Prevention, CHINA

## Abstract

**Background:**

Sparganosis is a neglected but important food-borne parasitic zoonosis. Clinical diagnosis of sparganosis is difficult because there are no specific manifestations. ELISA using plerocercoid crude or excretory–secretory (ES) antigens has high sensitivity but has cross-reactions with other helminthiases. The aim of this study was to characterize *Spirometra erinaceieuropaei* cysteine protease (SeCP) and to evaluate its potential application for serodiagnosis of sparganosis.

**Methodology/Principal Findings:**

The full length SeCP gene was cloned, and recombinant SeCP (rSeCP) was expressed and purified. Western blotting showed that rSeCP was recognized by the serum of sparganum-infected mice, and anti-rSeCP serum recognized the native SeCP protein of plerocercoid crude or ES antigens. Expression of SeCP was observed at plerocercoid stages but not at the adult and egg stages. Immunolocalization identified SeCP in plerocercoid tegument and parenchymal tissue. The rSeCP had CP activity, and the optimum pH and temperature were 5.5 and 37°C, respectively. Enzymatic activity was significantly inhibited by E-64. rSeCP functions to degrade different proteins and the function was inhibited by anti-rSeCP serum and E-64. Immunization of mice with rSeCP induced Th2-predominant immune responses and anti-rSeCP antibodies had the potential capabilities to kill plerocercoids in an ADCC assay. The sensitivity of rSeCP-ELISA and ES antigen ELISA was 100% when performed on sera of patients with sparganosis. The specificity of rSeCP-ELISA and ES antigen ELISA was 98.22% (166/169) and 87.57% (148/169), respectively (*P*<0.05).

**Conclusions:**

The rSeCP had the CP enzymatic activity and SeCP seems to be important for the survival of plerocercoids in host. The rSeCP is a potential diagnostic antigen for sparganosis.

## Author Summary

Sparganosis is a neglected tropical disease; its diagnosis is difficult and it is often misdiagnosed. ELISA using the crude or ES antigens of plerocercoids cross reacts with other helminthiases. Cysteine protease is a type of hydrolase and plays important roles in the development and survival of parasites; it has been used for diagnostic markers and vaccine targets for some parasitic diseases. In this study, a 36 kDa *Spirometra erinaceieuropaei* cysteine protease (SeCP) was expressed and purified. The results showed that SeCP was a plerocercoid stage-specific protein located in the teguments and parenchymal tissue. The rSeCP had cysteine protease activity and functioned to degrade host proteins. Vaccination of mice with rSeCP induced high levels of IgG1 and anti-rSeCP antibodies with the ability to kill plerocercoids in an ADCC assay. The rSeCP had a high sensitivity and specificity for detecting anti-plerocercoid antibodies, and could be used as a potential antigen for serodiagnosis of sparganosis.

## Introduction

*Spirometra erinaceieuropaei* (syn. *S*. *erinacei* or *S*. *mansoni*) is an intestinal tapeworm of wild and domesticated carnivores [1] and is most commonly seen in Asia, whereas *Spirometra mansonoides* is mainly found in North America [2]. Sparganosis is a zoonotic parasitic disease caused by the plerocercoids of the genus *Spirometra*. Plerocercoid infections in humans are acquired by ingesting raw or uncooked reptilian or amphibian flesh, placing frog or snake flesh on open lesions or inflamed areas, or drinking water contaminated with cyclops harboring procercoids. When the plerocercoid infects humans, it migrates widely in subcutaneous tissues and visceral organs where it undergoes no further development [3,4] and causes cutaneous and visceral larva migrans. In some cases, the larvae localize in vital organs or the central nervous system causing seizures, headache, blindness, epilepsy, paralysis, and even death. Ocular sparganosis is especially prevalent in China and Vietnam [5].

Sparganosis is one of a neglected tropical disease, but it is an important food-borne parasitic zoonoses. The diagnosis of sparganosis is very difficult and is often misdiagnosed because the larvae have no predilection for particular sites in the human body and specific clinical manifestations are lacking. A diagnosis of subcutaneous sparganosis can be made by detection of the larvae in a biopsy specimen, but a definitive diagnosis is very difficult for visceral and cerebral sparganosis as the larvae can be found only by surgical removal [6]. ELISA with crude or excretory–secretory (ES) plerocercoid antigens has high sensitivity for the detection of anti-plerocercoid antibodies, but the main disadvantage is the existence of an early “blind window” of time that results in false negatives during the early stages of infection and cross-reactions with serum samples from patients with other helminthiases (cysticercosis, paragonimiasis, clonorchiasis, etc.) [7,8]. Furthermore, the preparation of crude or ES plerocercoid antigens for ELISA requires the collection of plerocercoids from naturally infected hosts or experimentally infected laboratory animals, which is practically inconvenient in terms of cost, labor, and time. Recombinant proteins can be produced easily in large amounts by using the *in vitro* expression system and can be used as a good alternative to the crude or ES antigens in a standardized ELISA for serodiagnosis of sparganosis. Hence, studies on the sensitive and specific recombinant plerocercoid antigens will improve the early diagnosis and subsequent treatment of the disease.

Cysteine protease (CP) is a type of protein hydrolase that has cysteine residues in the active center of the enzyme and plays a principal role in the development and survival of parasites. CP has been used as a diagnostic marker and vaccine target for some parasitic diseases because of their immunogenicity [9]. Purified native or recombinant CP has been used for the diagnosis of sparganosis [10], schistosomiasis [11], fascioliasis [12], clonorchiasis [13], paragonimiasis [14] and ascariasis [15]. CP with different molecular weights (53, 36, 27 or 21 kDa) has been found in *S*. *erinaceieuropaei* plerocercoid soluble antigens [16–18]. The 36 kDa protein is the main antigenic component of plerocercoid ES proteins [19]. Some plerocercoid CP have been identified, and their biochemical properties and biological roles have been identified [10,20,21].

In our previous studies, *S*. *erinaceieuropaei* CP (SeCP, BAB62816, GI:15146346) was identified from the crude and ES proteins of *S*. *erinaceieuropaei* plerocercoids by two-dimensional electrophoresis (2-DE) and Western blotting combined with MALDI- TOF/TOF-MS [22,23]. The structure and function of SeCP were predicted using bioinformatics. The results showed that SeCP was a type of proteolytic enzyme with a variety of biological functions, and its gene sequence was 1 053bp length with the largest ORF at 1 011bp encoding 336 amino acids. SeCP contained a signal peptide, a complete cathepsin propeptide inhibitor domain, and a peptidase_C1A conserved domain located outside the membrane. No transmembrane domain was predicted. The secondary structure prediction for SeCP showed that there were 8 α-helixes, 7 β-strands, and 20 coils. *S*. *erinaceieuropaei* had the closest evolutionary status to *S*. *mansonoides* based on the SeCP phylogenetic analysis. SeCP had 15 potential antigenic epitopes and 19 HLA-I restricted epitopes, and it might be a potential diagnostic antigen for sparganosis [24].

The aim of this study was to express and characterize SeCP encoding a 36 kDa protein (BAA09820,GI:1834307) and to evaluate its potential application in the serodiagnosis of sparganosis.

## Methods

### Ethics statement

This study was carried out in accordance with the National Guidelines for Experimental Animal Welfare (MOST of People’s Republic of China, 2006). All animal procedures and the use of the patients’ serum samples in this study were approved by the Life Science Ethics Committee of Zhengzhou University (no. 2011–016). Before the patients’ serum samples were used, oral informed consent was obtained from all individuals.

### Parasites and animals

Plerocercoids were obtained from subcutaneous tissues and muscles of naturally infected frogs, which were collected from Henan province, China. The morphological and molecular analysis showed that all plerocercoid isolates in Henan province belonged to *S*. *erinaceieuropaei* plerocercoids. The plerocercoids were maintained by serial passage in BALB/c mice every 10–12 months. Adult *S*. *erinaceieuropaei* worms were collected from the small intestines of cats experimentally infected with plerocercoids at 2 months post infection [25,26]. The adults were washed with cold physiological saline disposed by DEPC. The samples were used immediately for RNA preparation or stored in liquid nitrogen until use.

Specific pathogen free (SPF) female BALB/c mice aged 5–6 weeks were used for the immunological studies. All mice were purchased from the Experimental Animal Center of Henan province (Zhengzhou, China).

### Experimental infection and serum samples

Ten female BALB/c mice were orally inoculated with 3 plerocercoids, and blood samples were collected when euthanized at 4 weeks post infection. The pre-infection serum samples from mice were used as a negative control. The serum samples of patients with sparganosis, echinococcosis, cysticercosis, schistosomiasis, paragonimiasis, clonorchiasis or trichinellosis were collected from our department. These patients were confirmed by a positive parasitological examination or specific serum antibodies. Healthy person sera were obtained from normal healthy individuals who were negative for serum specific antibodies of the above-mentioned parasitic diseases.

### Preparation of crude antigens and ES antigens

Crude antigens and ES antigens from *S*. *erinaceieuropaei* plerocercoids were prepared as described previously [22,23,27], and the protein concentration was determined by the Bradford method at 4.2 mg/ml and 1.04 mg/ml, respectively.

### Cloning, expression, and identification of SeCP

The full-length cDNA sequence of the SeCP gene was obtained from GenBank (D63670, protein is BAA09820, GI:1834307) and analyzed by bioinformatics [24]. SeCP has a signal peptide with 19 amino acids. While designing the specific primers, the N-terminal signal peptide sequence was omitted and the SeCP molecular weight was approximately 36 kDa. The SeCP gene was amplified by nested PCR. First, the target cDNA underwent the first run of PCR with the first set of primers (forward, 5' TAGGATGAAGTTCGTAATATACGTTGCC 3'; reverse, 5'-CCAAA AGATGTTTATTTACTCCACAGGTG-3’), and the cycling protocol was as follows: 28 cycles of 94°C for 1 min, 50°C for 45 s and 72°C for 1 min. The products underwent a second run with the second set of specific primers carrying *EcoR*Iand *Hind* III restriction enzyme sites (bold and italicized) (forward, 5’-CG***GAATTC***TCGACTGAA AGTGAGACGTACGTCC-3’ and 5’- CCC***AAGCTT***TTACACG GTTGGATAGCTTGCCAT -3’), and the cycling protocol was as follows: 28 cycles of 94°C for 1 min, 56°C for 45 s and 72°C for 1 min. The final PCR products were purified, digested, and cloned into the pGEM-T vector (Promega, USA) and subsequently sub-cloned into the expression vector pMAL-c2X (New England Biolabs, USA). The recombinant plasmid was then transformed into *Escherichia coli* TB1 (New England Biolabs, USA). Expression of rSeCP was induced by adding 0.1 mM IPTG at 30°C for 3 h. The rSeCP was purified by Amylose Pre-packed Column (NEB Ltd, China) and identified by SDS-PAGE. Images of gels were recorded using ImageScanner (GE Healthcare, Fairfield, CT). Another gel was prepared by the same method and used for the Western blotting analysis described below.

### Generation and determination of antibodies to rSeCP

Thirty BALB/c mice were randomly divided into three groups of ten animals each. The immune mice were subcutaneously vaccinated with 20 μg of purified rSeCP with an equal volume of complete Freund’s adjuvant followed by three boosts with the same dose of the incomplete adjuvant at 10-day intervals [28]. The control group of mice was injected only with adjuvant or PBS using the same immunization schedule. Approximately 50 μl of tail blood were collected at 0, 10, 20, 30, and 40 days post-immunization from the vaccinated mice. The specific anti-rSeCP antibody levels of IgG and its subtype (IgG1 and IgG2a) in serum samples of immunized mice were assayed using ELISA with rSeCP (2.5 μg/ml) as described previously [29].

### Antigenicity analysis of rSeCP by western blotting

The purified rSeCP and the crude and ES plerocercoid antigens were separated by SDS–PAGE (12% gel) and transferred onto nitrocellulose (NC) membranes (Millipore, USA) at 18 V for 35 min by semi-dry transfer cell (Bio-Rad, USA). Western blotting of rSeCP antigenicity was performed as described previously [25,30].

### RT-PCR analysis of SeCP gene transcription

To observe transcription of the SeCP gene at different *S*. *erinaceieuropaei* developmental stages, total RNA was extracted from the plerocercoids of frogs or mice and the adults from cats. RT-PCR was performed as previously described [28] and the primers specific to SeCP were the first set of primers described above. As an internal control, *S*. *erinaceieuropaei* glyceraldehyde-3- phosphate dehydrogenase (GAPDH, GenBank accession No. AB031067.1) was used as the housekeeping gene for this study and the PCR product size of the internal control was 670 bp. Amplified PCR products were analyzed on a 1% agarose gel. Negative control reactions, which contain all of the reagents except Milli-Q water being substituted for cDNA as the template, were included to ensure that the reaction system was not contaminated. The entire experiment was carried out three times.

### Immunofluorescence test (IFT)

IFT was used to locate the position of SeCP in the cestode. The tissue sections of *S*. *erinaceieuropaei* plerocercoids and adult worms was first retrieved after microwaving for 20 min with a 0.01 M citric acid buffer (pH 6.0), blocking with 5% normal goat serum in PBS, and then incubating at 37°C for 1 h with a 1:10 dilution of anti-rSeCP serum, serum of mice infected with plerocercoids, normal mouse serum or PBS. After washing three times in PBS, the sections were incubated with a 1:50 dilution of FITC-labeled anti-mouse IgG (Santa Cruz, USA), and the nuclei were stained with propidium iodide (PI) at 37°C for 15 min. After washing five times with PBS, the sections were examined under a fluorescent microscope (Olympus, Japan) [31].

### Substrate gel electrophoresis assay

The enzymatic activity of rSeCP was assayed by substrate gel electrophoresis with a 12% SDS–PAGE gel copolymerized with 0.1% gelatin as the substrate, as previously reported [32,33].

### Enzyme activity assays

The enzymic activity of rSeCP was also determined by cleavage of the fluorescent substrate Z-carbobenzoxy-L-phenylalanyl-L-arginine-(7-amino-4-methylcoumarin) (Z-Phe-Arg-AMC, Sigma) as previously described [34]. Briefly, the substrate was prepared at a concentration of 2 mM dissolved in dimethylsulfoxide (DMSO). The reaction was carried out in a volume of 240 μl of standard assay buffer (100 mM sodium acetate, 5 mM dithiothreitol (DTT); pH 5.5) coupled with an appropriate quantity of enzyme. The rSeCP was pre-incubated in assay buffer at a volume of 160 μl at 37°C for 30 min, while substrate at a final concentration of 3 μM was pre-incubated in assay buffer as well. The reaction was started by mixing the two samples together. The fluorescence intensity was continuously measured with spectrophotofluorometry (Synergy H1, BioTek, USA) using excitation and emission wavelengths of 355 nm and 460 nm, respectively. The pH activity profiles were established using the following buffers: 100 mM sodium acetate buffer (pH 3.0–5.5), 100 mM sodium phosphate (pH 6.0–7.5), and 100 mM Tris–HCl (pH 8.0–8.5) containing 5 mM DTT. The optimal temperature for rSeCP activity was assayed at 10°C, 20°C, 28°C, 37°C, 45°C and 50°C. The residual activity in the samples and the control (without reagents) was also determined. The highest enzyme activity was used as the control (100% of relative activity). The influence of metal ions on rSeCP activity was determined and different concentrations of Cu^++^, Mn^++^ and Zn^++^ metal ions (0.01 mM, 0.1 mM, 1 mM, 10 mM, and 100 mM) were added to the assay in the form of CuCl_2_, MnCl_2_ and ZnCl_2_, respectively. The relative fluorescence unit (RFU) was used to express catalytic activity. rSeCP was also pre-incubated with inhibitor at 37°C for 30 min. Substrate was added, and incubated at 37°C for 30 min. Inhibitors included PMSF, phenylmethylsulfonyl fluoride; AEBSF, 4-(2-Aminoethyl) benzenesulfonyl fluoride; EDTA, ethylenediaminetet-raacetate; E-64, and L-trans-epoxysuccinyl-leucylamide (4-guanidino) butane. Percentages are based on activity in the presence of 10 mM DTT without inhibitors.

### Degradation of different proteins by rSeCP

Rabbit immunoglobulin (Ig), human Ig (Laboratorios LANDERLAN. S.A. Spain), bovine hemoglobin (Hb), fibronectin, and bovine serum albumin (BSA, Sigma) were used to determine the hydrolytic function of rSeCP. Each protein (1 mg/ml) was incubated with rSeCP (1 μg) in 100 mM sodium acetate (pH 5.5) or 100 mM sodium phosphate (pH 7.0) containing 5 mM DTT at 37°C overnight. Each protein (1 mg/ml) was also incubated with different rSeCP concentrations (1, 2 or 3 μg) in 100 mM sodium acetate (pH 5.5). The inhibition of anti-rSeCP serum on SeCP catalytic activity was determined by using human Ig as the substrate for rSeCP activity as described previously [35]. For the inhibition assay, 3 μg of rSeCP was incubated with anti-rSeCP serum at 37°C for 1 h, following by incubation at 37°C overnight with the addition of 1 mg/ml final concentration of human Ig. The total volume of the mixture was 40 μl. Anti-rSeCP serum used in this assay was diluted at 1:50, 1:100, 1:500, and 1:1000, respectively. The inhibitor E-64 was also used to determine the enzymatic activity with the same method. After incubation, 5 μl of each sample was analyzed with 12% SDS–PAGE as described above [36].

### *In vitro* antibody-dependent cellular cytotoxicity (ADCC) assay

To determine the cytotoxic effects of anti-rSeCP antibodies to plerocercoids an *in vitro* ADCC assay was performed as described previously [37]. Sera from plerocercoid-infected mice were used as the positive control, normal mouse sera served as negative controls, and PBS as the blank control. Briefly, the ADCC assay was performed by adding 10 plerocercoids to a suspension of 2×10^5^ mouse peritoneal macrophages (MPM) collected from normal BALB/c mice. Sera were diluted at 1:10 with RPMI 1640 media and added to the suspension to make a final volume of 2 ml. The suspension was then added to 6 well culture plates and incubated at 37°C and 5% CO_2_ for 48 h. Plerocercoid viability was identified by the morphology and activity under a light microscope at different time intervals after incubation. Worms that were limp or straight without movements were counted as non-viable and disintegrated worms were counted as dead. The live larvae were active and wriggling [37,38]. Results were expressed as the ratio of immobile or dead parasites to the total number of parasites recovered within each experiment.

### ELISA

ELISA with rSeCP (rSeCP-ELISA) and ES antigens (ES-ELISA) were performed as previously described [27]. Briefly, the ELISA plates (Corning, USA) were coated with rSeCP (2.5 μg/ml) and ES proteins (2.5 μg/ml) in 100 μl of bicarbonate buffer (pH 9.6) at 4°C overnight. After blocking with 5% skim milk in PBST at 37°C for 1 h, the following reagents were sequentially added and incubated at 37°C for 1 h as follows: (1) sera diluted at 1:100 in PBST, and (2) HRP-conjugated anti-human IgG (Sigma, USA) diluted at 1: 5 000. After the final wash, the reactions were detected by the substrate ortho-phenylene diamine (OPD, Sigma, USA) plus H_2_O_2_ and stopped with 50 μl/well of 2 M H_2_SO_4_. Optical density (OD) values at 490 nm were determined with a microplate reader (TECAN, Austria). All samples were run in duplicate. Test sera/negative serum OD values <2.1 were regarded as negative and those ≥2.1 as positive. The cut-off values of rSeCP-ELISA and ES-ELISA were 0.34 and 0.42, respectively.

### Statistical analysis

All statistical analyses were performed with SPSS for Windows, version 17.0 (SPSS Inc., Chicago, IL). The chi-square test and repeated measures of analysis of variance (ANOVA) were used to determine the difference among the groups at various periods. Intra- and intergroup statistical analyses were performed with one-way ANOVA (LSD test). The statistical significance was defined as *P* < 0.05.

## Results

### Expression and purification of rSeCP

**Fig 1:**
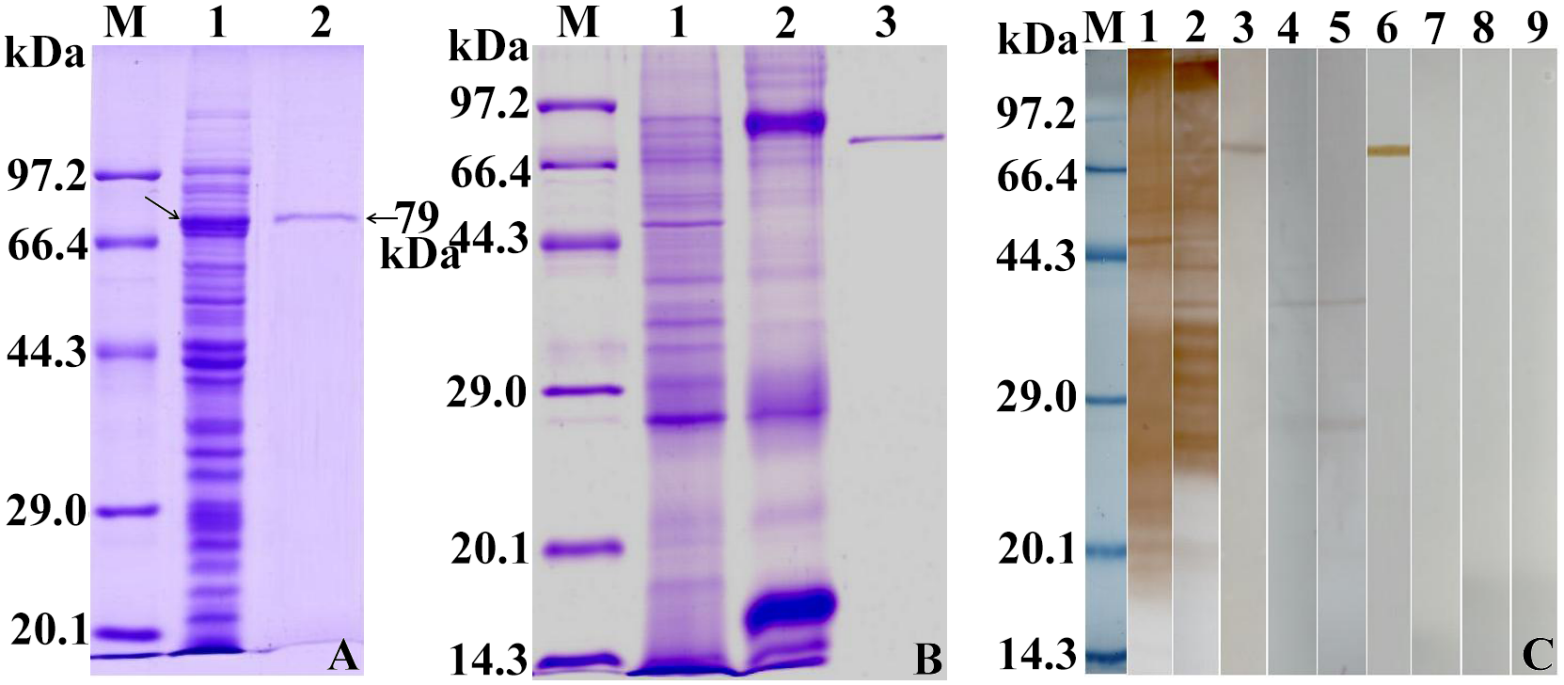
SDS-PAGE and Western blotting analysis of rSeCP antigenicity. (A) SDS-PAGE analysis of rSeCP. M, protein molecular weight marker; lane 1, the lysis of the induced recombinant bacteria harboring pMAL-c2X-SeCP; lane 2, rSeCP purified by Amylose Pre-packed Column. The recombinant MBP fusion protein is marked with an arrow (→). (B) SDS-PAGE analysis of plerocercoid crude (lane 1) and ES antigens (lane 2) and rSeCP (lane 3). (C) Western blotting analysis of rSeCP antigenicity. The plerocercoid crude antigens (lane 1), ES antigens (lane 2), and rSeCP (lane 3) were recognized by sera of mice infected with plerocercoids. The native SeCP protein in crude antigens (lane 4) and ES antigens (lane 5) and rSeCP protein (lane 6) were recognized by anti-rSeCP serum. The plerocercoid crude antigens (lane 7), ES antigens (lane 8), and rSeCP (lane 9) were not recognized by sera of normal mice.

The sequenced SeCP gene cloned in this study was 99.42% identical to the SeCP gene sequence in GenBank (D63670.1) and had an active site containing the classic catalytic triad, Cys-His-Asn triad (Cys145, His283, Asn303). The coding sequence of the SeCP gene was cloned into the prokaryotic expression vector pMAL-c2X. After induction with 0.1 mM IPTG, TB1 bacteria harboring the recombinant plasmid pMAL-c2X-SeCP expressed a soluble fusion protein. On SDS-PAGE analysis, the molecular size of rSeCP was 79 kDa and consistent with the predicted combined size of the protein encoded by the cDNA clone (36 kDa) and maltose-binding protein (MBP) tag from the vector (43 kDa) (Fig 1A). The concentration of rSeCP was 0.42 mg/ml.

### Western blotting analysis of rSeCP antigenicity

A Western blotting analysis showed that rSeCP was recognized by the serum of mice infected with plerocercoids and anti-rSeCP serum (Fig 1C). Anti-rSeCP serum recognized the native proform SeCP protein with 36 kDa of crude and ES plerocercoid proteins. Additionally, another approximately 27 kDa band of crude and ES plerocercoid proteins, which might be the mature form of the 36 kDa SeCP, was also weakly recognized by anti-rSeCP serum. The results indicated that SeCP is one component from both the crude and ES proteins of plerocercoids.

### RT-PCR analysis

**Fig 2:**
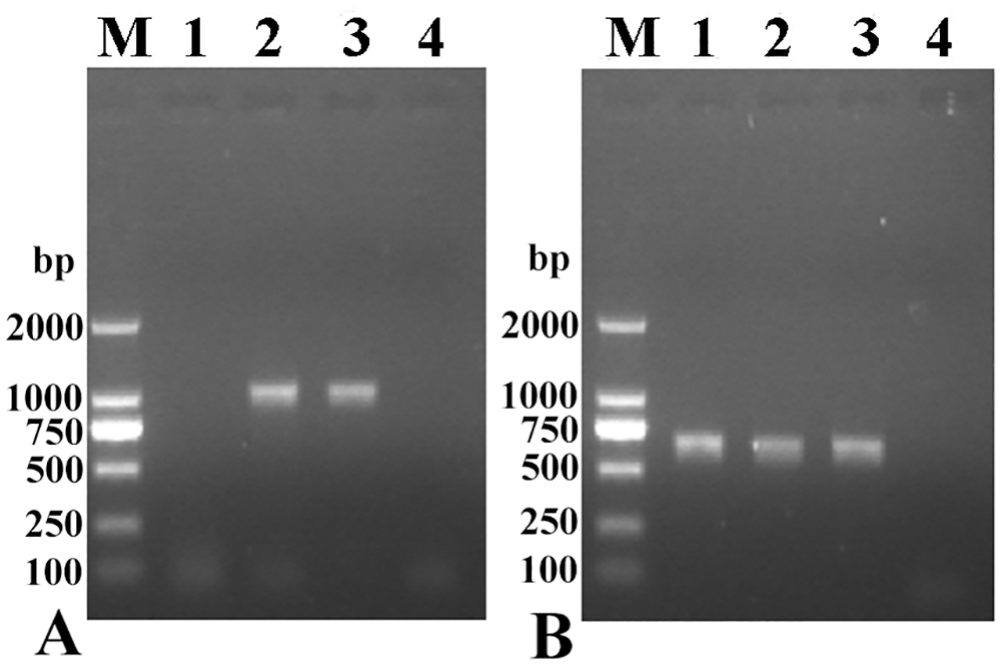
RT-PCR analysis of SeCP gene transcription at different *S*. *erinaceieuropaei* stages. RT-PCR detection of mRNA transcription for SeCP gene (A) and GAPDH gene (B) at different developmental stages of *S*. *erinaceieuropaei*. M, DNA marker; lane 1, adults; lane 2, plerocercoids collected from experimentally infected mice; lane 3, plerocercoids from naturally infected frogs; line 4, MilliQ H_2_O control.

The mRNA transcription (1045 bp) for the SeCP gene was observed at different plerocercoid stages from frogs or mice. Furthermore, the primers for a standard gene (GAPDH) generated the expected size band (670bp) in all of the samples (Fig 2).

### Expression and immunolocalization of SeCP at different stages

**Fig 3:**
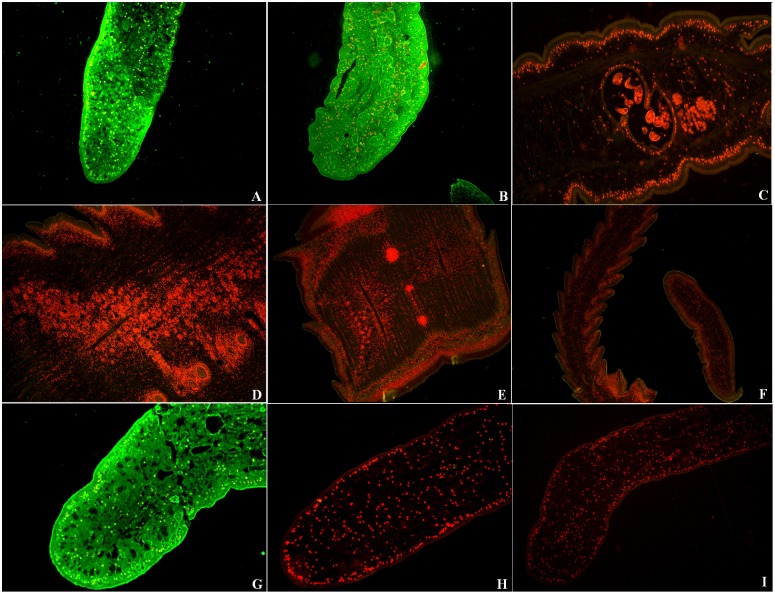
Expression and immunolocalization of SeCP at different developmental stages of *S*. *erinaceieuropaei*. The results of IFT with the sections of *S*. *erinaceieuropaei* plerocercoids and adult worms reacted with anti-rSeCP serum. The immunostaining is seen at the teguments and parenchymal tissues of plerocercoids from mice (A) and plerocercoids from frogs (B), but no immunostaining was observed in the sections of gravid and eggs (C), mature (D), immature (E) proglottids, and scolex and neck (F) of adult worm. The plerocercoid reacted with serum of mice infected with plerocercoids as a positive control (G), and did not show recognition by normal mouse serum (H) and PBS (I) as a negative control.

Immunolocalization showed that specific fluorescent staining was observed in tegument and parenchymal tissue of plerocercoid collected from infected mice and frogs using anti-rSeCP serum, but no fluorescent staining was detected in the adult worm and egg sections (Fig 3).

### rSeCP enzyme activity

The protease activity of purified rSeCP was investigated using a gelatin SDS-PAGE assay. When the gels were incubated in an acidic buffer (pH 5.5), a zone of hydrolysis was visible, and the successful inhibition of enzymatic activity with E-64, a specific cysteine protease inhibitor, demonstrated that purified rSeCP was the expected cysteine protease.

**Fig 4:**
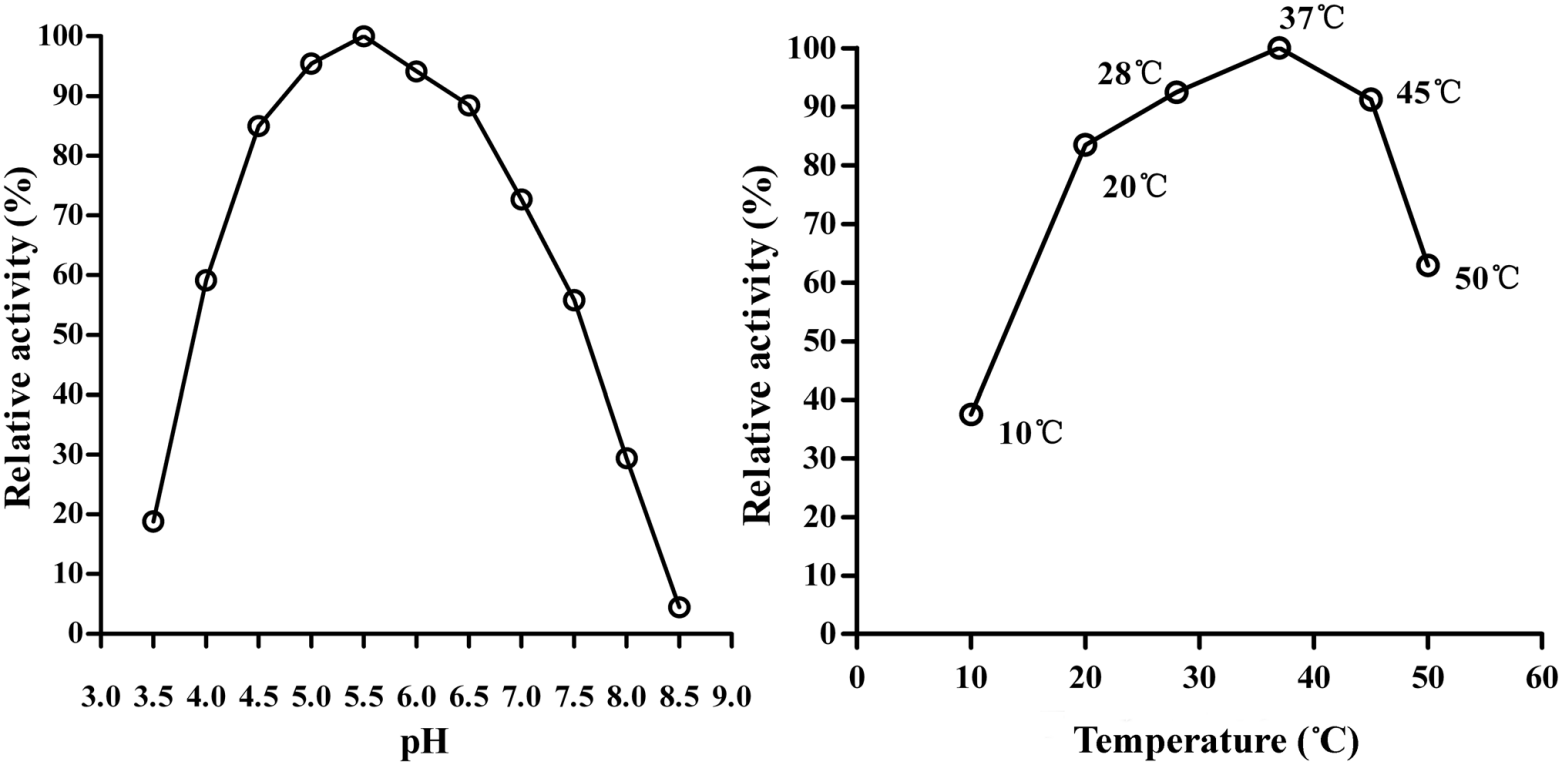
Optimum pH (A) and temperature (B) of rSeCP enzyme activity. Enzyme activities were assayed in 100 mM sodium acetate (pH 3.5–5.5), sodium phosphate (pH 6.0–8.5), in the presence of 1 mM DTT. Maximal activity was shown as 100%.

**Fig 5:**
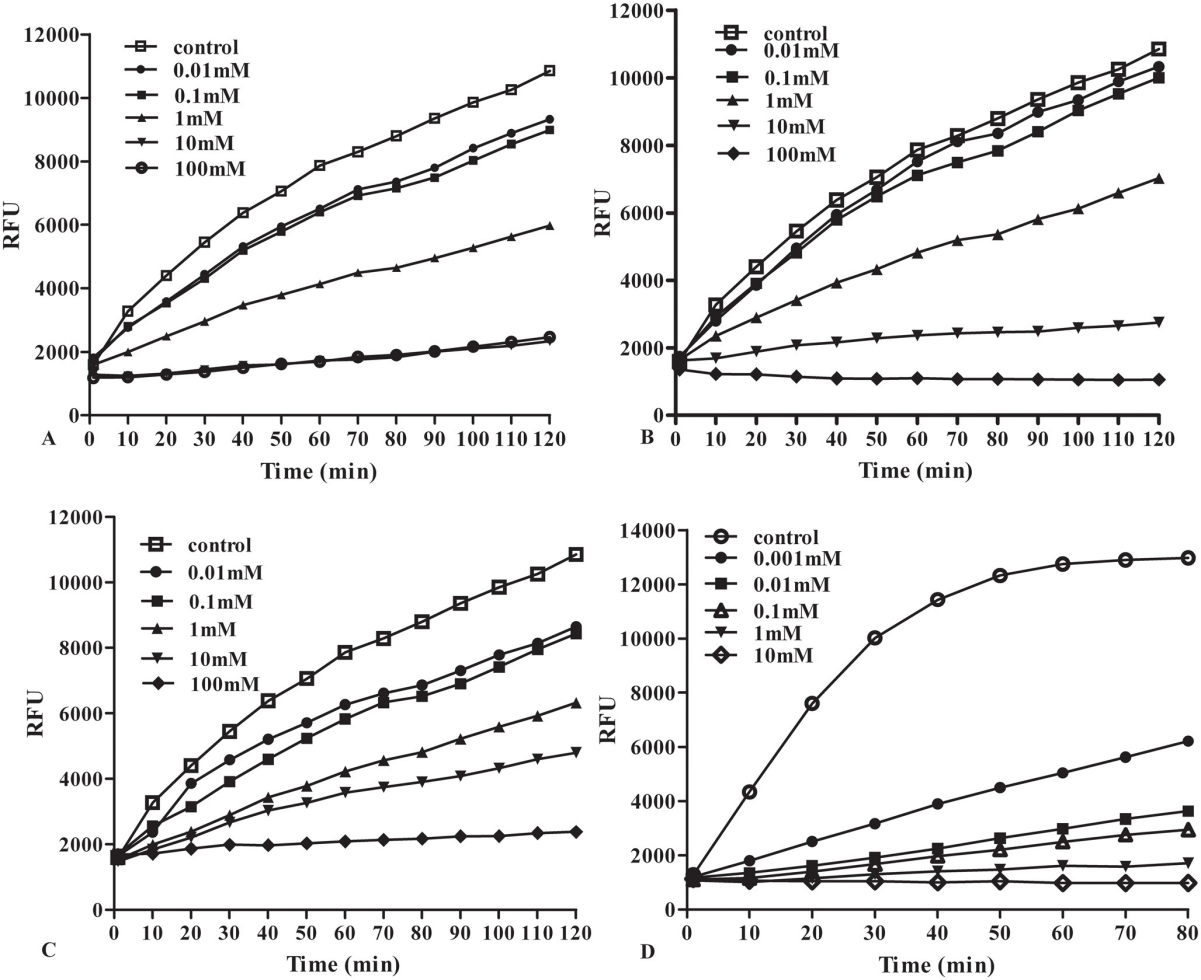
The effect of different concentrations of metal ions and E-64 on the progress curve of the enzymatic reaction catalysed by rSeCP, expressed as relative fluorescence unit (RFU). A, Cu^++^; B, Zn^++^; C, Mn^++^; D, E-64.

To further confirm the proteolytic activity of rSeCP, the conventional enzyme assay was carried out with the use of fluorogenic peptide substrates. rSeCP showed a broad pH range, from 3.5 to 8.5, and maximum activity was detected at pH 5.5. The optimum temperature was 37°C (Fig 4). When added to the assay environment in the form of chlorides, rSeCP catalytic activity was inhibited by irons (Cu++, Mn++ and Zn++) (Fig 5). Under the same conditions, rSeCP enzymatic activity could be significantly inhibited only by E-64, which is known to inhibit cysteine proteases at the concentrations commonly used in these assays (Table 1). The inhibition of rSeCP catalytic activity by irons and E-64 was dose-dependent.

**Table 1:** Effects of endopeptidase inhibitors on rSeCP.

Inhibitors*	%Activity
None	100.00
Serine class	
PMSF (1 mM)	100.20
AEBSF (1 mM)	84.20
Metallo class	
EDTA (10 mM)	66.70
1,10-Phenanthrolin (1 mM)	67.50
Cysteine class	
E-64 (5μM)	0.80

* PMSF, phenylmethylsulfonyl fluoride; AEBSF, 4-(2-Aminoethyl) benzenesulfonyl fluoride; EDTA, ethylenediaminetet-raacetate; E-64, L-trans-epoxysuccinyl-leucylamide (4-guanidino) butane. Percentages based on activity in the presence of 10mM dithiothreitol (DTT) without inhibitors. All assays were done in duplicate.

### rSeCP degradation of various proteins

**Fig 6:**
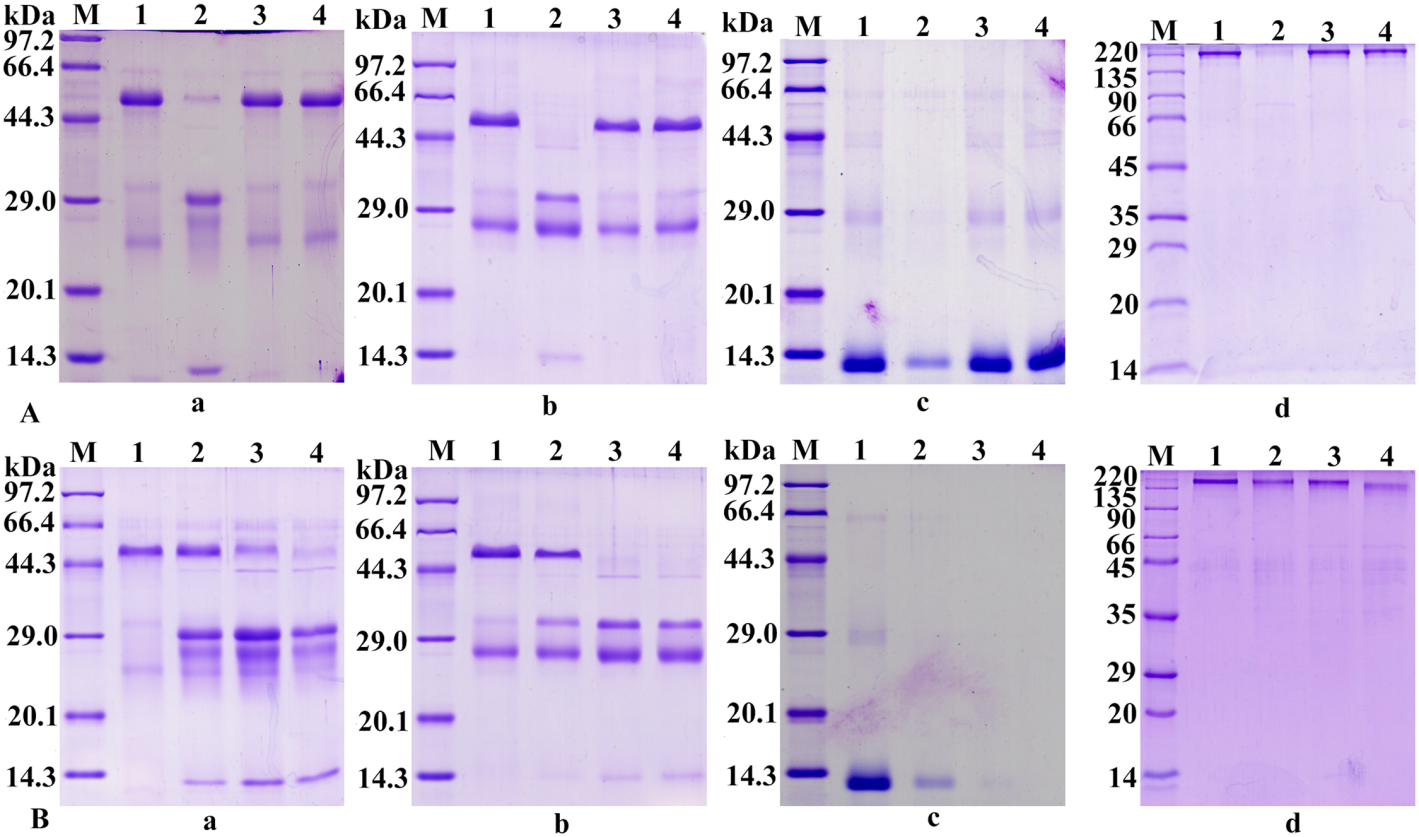
rSeCP degradation of various proteins. (A) Degradation of rabbit Ig (a), human Ig (b), bovine Hb (c) and fibronectin (d) by rSeCP at pH 5.5 and pH 7.0, respectively. lane M, protein molecular weight marker; lane 1, protein alone at pH 5.5; lane 2, protein incubated with rSeCP at pH 5.5; lane 3, protein alone at pH 7.0; lane 4, protein incubated with rSeCP at pH 7.0. (B) Degradation of rabbit Ig (a), human Ig (b), bovine Hb (c) and fibronectin (d) by the different concentration of rSeCP at pH 5.5. lane M, protein molecular weight marker; lane 1, protein alone; lanes 2–4, protein incubated with 1, 2 and 3 μg of rSeCP.

**Fig 7:**
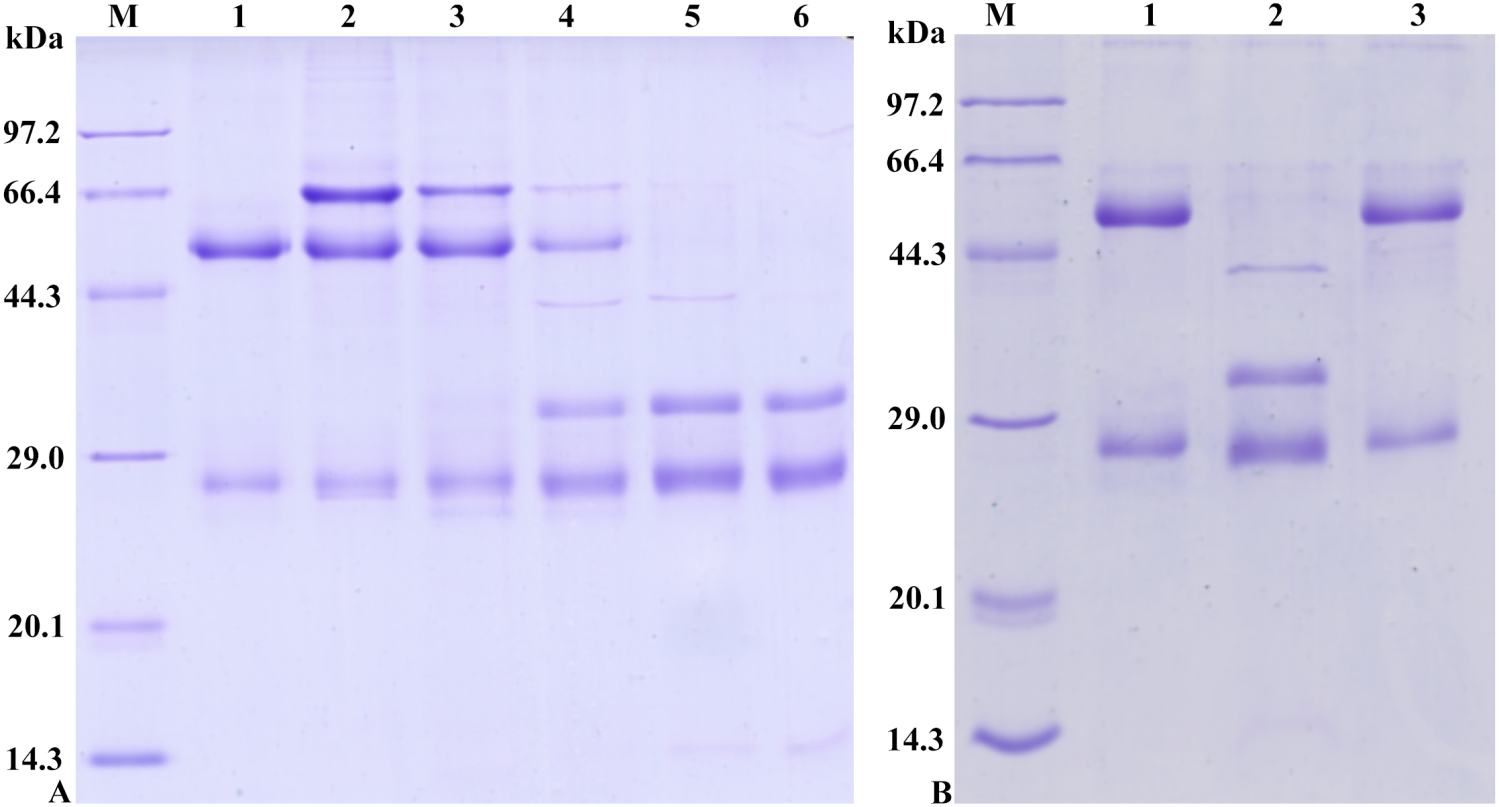
Inhibition of rSeCP enzymatic activity. (A) Degradation of human Ig by rSeCP was neutralized by anti-rSeCP serum. lane M, protein molecular weight marker; lane 1, human Ig alone; lanes 2–5, anti-rSeCP serum diluted at 1:50, 1:100, 1: 500, and 1:1000 with 100 mM sodium acetate (pH 5.5) containing 5 mM DTT; lane 6, rSeCP + human Ig. (B) Degradation of human Ig by rSeCP was inhibited by E-64. lane M, protein molecular weight marker; lane 1, human Ig alone; lane 2, rSeCP + human Ig; lane 3, rSeCP +E-64 + human Ig. Anti-rSeCP serum or the inhibitor E-64 (10 μM) was firstly incubated with rSeCP at 37°C for 2 h, followed by another incubation at 37°C overnight, with adding 1 mg/ml final concentration of human Ig.

To investigate the putative biological roles of rSeCP, the proteolytic activity of rSeCP against several natural substrate proteins including Rabbit Ig, mouse Ig, bovine Hb, fibronectin and BSA was assayed. The results showed that the proteins were readily hydrolyzed by rSeCP at an acidic pH of 5.5, but no degradation was observed at a neutral pH of 7.0 (Fig 6A). BSA showed no hydrolysis at acidic and neutral pHs. Furthermore, when different concentrations of rSeCP were used to test its proteolytic activity, the result showed that the proteins were degraded more thoroughly with an increase in rSeCP (Fig 6B). Evaluation of the inhibition of rSeCP catalytic activity by anti-rSeCP antibodies was determined using human Ig as the substrate for rSeCP activity. The results showed that the catalytic activity of rSeCP was inordinately inhibited by different dilutions of anti-rSeCP sera, while the catalytic activity was effectively inhibited by E-64 (Fig 7).

### Humoral immune responses induced by vaccination with rSeCP

**Fig 8:**
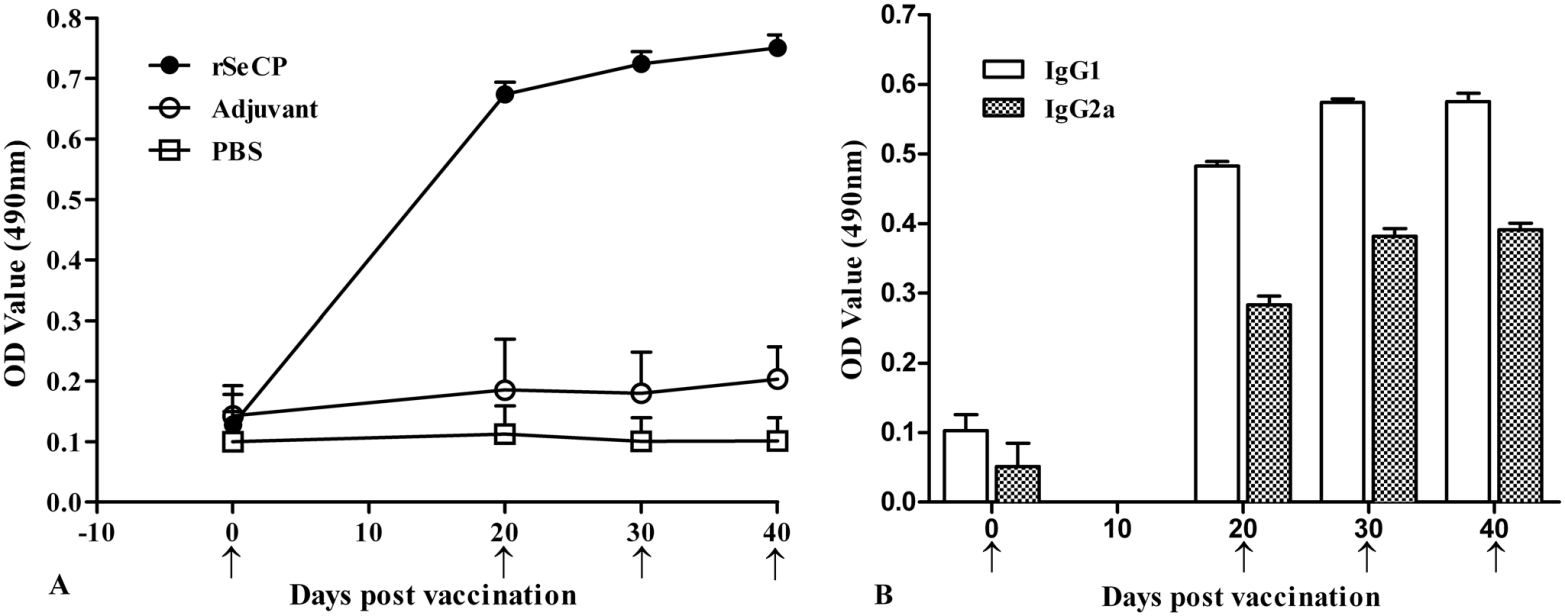
Analysis of antibody responses in mice immunization with rSeCP. (A) Anti-rSeCP IgG levels in the sera of immunized mice were measured by ELISA. (B) The IgG subclass (IgG1 and IgG2a) responses in immunized mice were detected at different time point post vaccination. The OD values shown for each group are the mean ± SD of the antibody levels. The immunization time points are marked with an arrow (↑).

The specific IgG antibodies to rSeCP in the serum of immunized mice were determined by ELISA using rSeCP as antigens, and the IgG antibody titer of anti-rSeCP serum was 1:10^6^, suggesting that rSeCP has good immunogenicity. Anti-rSeCP IgG levels in mice immunized with rSeCP were greatly increased following the first and second immunization (Fig 8). However, none of the mice vaccinated with adjuvant or PBS showed a significantly detectable specific anti-rSeCP antibody responses. The results of the IgG subclass antibody assay showed that after the second and third immunization, the levels of IgG1 were significantly higher than that of IgG2a (t_20d_ = 52.043, t_30d_ = 61.425, *P*<0.01).

### *In vitro* ADCC

**Fig 9:**
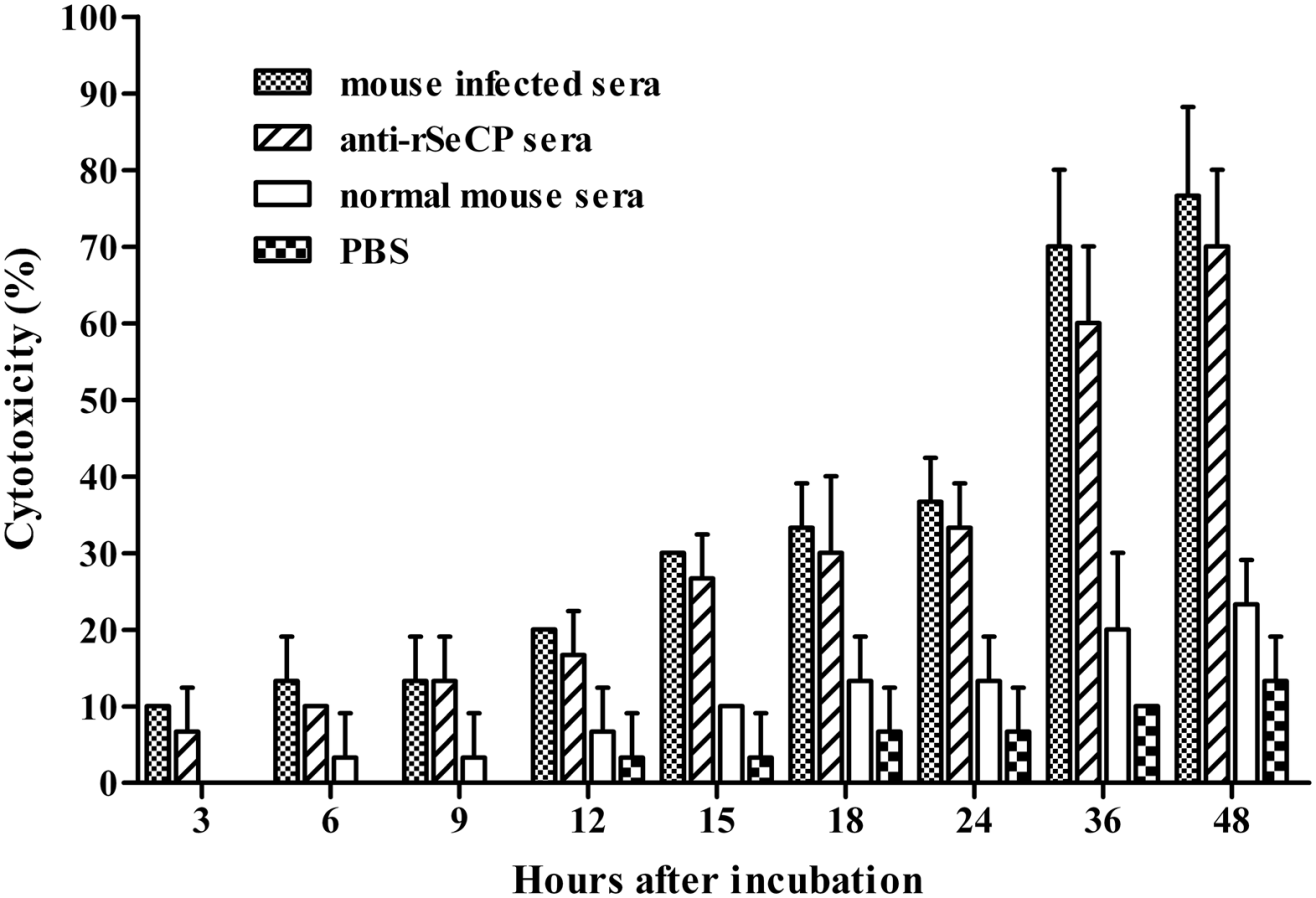
Results of antibody-dependent cellular cytotoxicity (ADCC) assay against spargamum.

The ADCC results showed that anti-rSeCP serum promoted adherence of MPM to plerocercoids and the cytotoxicity increased with longer incubation times in the infection serum and anti-rSeCP serum groups (*F* = 86.408, *P* <0.05) (Fig 9). At 48 h after incubation, anti-rSeCP serum significantly induced plerocercoid death (70.00% cytotoxicity) compared to plerocercoids incubated with normal mouse sera (25.33%, *P* <0.05) and PBS control (13.33%, *P*<0.05), while there was no significant difference compared to mouse infection sera (76.67%, *P*>0.05) (S1 Table).

### Detection of serum anti-plerocercoid IgG antibodies in patients with sparganosis

The sensitivity of both rSeCP-ELISA and ES-ELISA for detecting the serum samples of patients with sparganosis was 100% (20/20) (Table 2); however, the specificity of rSeCP-ELISA for detecting sera of patients with cysticercosis, echinococcosis, schistosomiasis, paragonimiasis, clonorchiasis and trichinellosis, and healthy persons was significantly greater than the specificity of ES-ELISA at 98.22% (166/169) and 87.57% (148/169) (*χ* ^*2*^ = 22.604, *P*<0.01), respectively.

**Table 2:** Detection of anti-plerocercoid IgG antibodies in serum samples of patients with sparganosis and other parasitosis by rSeCP-ELISA and ES-ELISA.

		rSeCP-ELISA		ES-ELISA	
Sera of patients with	No. of serum samples	OD values ($\bar{x}\pm S$)	No. of positive serum samples (%)	OD values ($\bar{x}\pm S$)	No. of positive serum samples (%)
**sparganosis**	20	0.55±0.07	20 (100.00)	0.68±0.05	20 (100.00)
**echinococcosis**	20	0.20±0.07	0 (0)	0.23±0.14	3 (15.00)
**cysticercosis**	20	0.16±0.10	1 (5.00)	0.14±0.11	1 (5.00)
**clonorchiasis**	7	0.17±0.04	0 (0)	0.29±0.15	2 (28.57)
**schistosomiasis**	20	0.17±0.07	1 (5.00)	0.18±0.12	2 (10.00)
**paragonimiasis**	20	0.19±0.08	1 (5.00)	0.41±0.17	13 (65.00)
**trichinellosis**	22	0.15±0.07	0 (0)	0.12±0.08	0 (0)
**healthy persons**	60	0.16±0.06	0 (0)	0.20±0.08	0 (0)

## Discussion

Cysteine protease is a candidate diagnostic antigen with good specificity for some helminth infections, including *Angiostrongylus cantonensis* [33], *Necator americanus* [39], *Toxocara canis* [15], *Paragonimus westermani* [14], *Schistosoma japonicum* [40], *Clonorchis sinensis* [41], *Taenia solium* [42], and *S*. *erinaceieuropaei* [17]. The *E*. *coli* prokaryotic expression system has been considered as the most fully explored and commonly used system for gene engineering to produce recombinant proteins due to its security, simplicity, rapidity and high efficiency. In this study, SeCP encoding a 36 kDa protein from *S*. *erinaceieuropaei* plerocercoids was successfully cloned and expressed using the MBP fusion-based pMAL-c2X vector system, which contained a mutation in the translocation signal and thus would yield only cytoplasm-associated recombinant proteins [43]. The recombinant fusion protein was highly soluble in the bacteria’s cytoplasm and had good immunogenicity in mice; therefore, it could be used as an immunogen to produce antibodies after purification by affinity chromatography with amylose resin. Our results showed that the purified rSeCP induced a strong specific humoral immune response in immunized BALB/c mice, and the specific IgG antibody titer of anti-rSeCP serum was 1:10^6^ as determined by rSeCP-ELISA.

On Western blotting analysis, anti-rSeCP serum strongly recognized the native 36 kDa SeCP protein of plerocercoid crude or ES antigens. The results demonstrated that SeCP was a component of both the crude and ES antigens of *S*. *erinaceieuropaei* plerocercoids. Meanwhile, another band of approximately 27 kDa in plerocercoid crude and ES antigens was also weakly recognized by anti-rSeCP serum. The results suggested that the 36 kDa SeCP protein is an inactive proform while the 27 kDa protein is an enzymatically active mature form. Previous studies showed that an approximately 28 kDa SeCP from the plerocercoid crude and ES antigens, which was purified by ion-exchange chromatography and thiopropyl-Sepharose, had cysteine protease enzymatic activity and was consistent with the mature form of the 35 kDa protein [17]. The molecular weight of purified native SeCP was very similar to those of the proteins recognized by anti-rSeCP serum in crude and ES antigens in our study. The molecular weight difference might be the result of subjective analysis and judgment of these protein bands. Another study also demonstrated that the 27 kDa SeCP purified from crude plerocercoid antigens had cysteine protease activity [44].

The results of the RT-PCR analysis and IFT indicated that SeCP was expressed in the plerocercoid stage of different hosts (frogs and mice) but not in the adult worm and egg. A previous study showed that a 27 kDa cathepsin L-like cysteine protease was expressed in the coracidium and plerocercoid stages of *S*. *erinaceieuropaei* but not in immature eggs and adults [44]. These results suggest that the 27 kDa cysteine protease is only expressed in the invasive and migratory stages of the parasite. Our results also demonstrated that SeCP is the plerocercoid-specific protein. Expression of different types of cysteine proteases in different developmental stages has been observed in other parasites, including *Schistosoma* and *Fasciola hepatica* [32,45], suggesting that these cysteine proteases may have stage-specific functions, such as degrading different tissue barriers or contrasting protein composition. For the intracellular localization of SeCP, the bioinformatics prediction results indicated that the SeCP was cathepsin L, which is a lysosomal cysteine protease that plays a major role in intracellular protein catabolism [24], suggesting that SeCP might be located in the lysosomes or secretory granules of cells. The location of 36 and 29 kDa proteins in *S*. *erinaceieuropaei* plerocercoids were analyzed by immunohistochemical staining using serum from mice immunized with crude plerocercoid extract, and the two proteins were located in the syncytial tegument, tegumental cells, muscles and parenchymal cells, and lining cells of excretory canals; when the monoclonal antibody reacting to the 36 and 29 kDa protein was used, and the two proteins were located in the syncytial tegument and tegumental cells [46]. In *Schistosoma mansoni*, the cathepsin B and Ll-like cysteine proteases were located in the cortex and the cecum intestinal epithelial cells of adult worms [47], while the cathepsin L2 in female and male adults existed in the reproductive system and the subcortical cortex cells of the gynecophoral canal [48]. The *Fasciola hepatica* cysteine protease was synthesized in the intestinal epithelial cells [49].

Previous studies demonstrated that many extracellular parasite proteases function to aid in the invasion of tissues and cells, hatch of eggs or evasion of host immune system. Cysteine protease is the major factor in parasitic pathogenicity because it induces tissue damage and facilitates invasion or enables the parasites to salvage metabolites from host proteins [50]. It has been shown that *S*. *erinaceieuropaei* plerocercoids secrete plentiful cysteine proteases [51].

The results of gelatin substrate gel digestion showed that rSeCP had CP enzymatic activity, possibly because the recombinant inactive proform enzyme was refolded and converted to an enzymatically active mature form after being fused with MBP in *E*. *coli* by altering the inhibitor domain and exposing the active site. It is also possible that because the inactive proform enzyme contained the mature domain and leader peptide, which is a type of effective enzyme inhibitor, the leader peptide and the mature domain were combined together tightly in the neutral environment but were separated rapidly in the acidic environment and the proform was converted into the enzymatically active mature form [52,53]. However, the activation mechanism of an inactive 36 kDa proform converted to the enzymatically active 27 kDa mature form is not well known. The optimum pH for rSeCP protease activity in acidic conditions suggested that the rSeCP was specially fit for enzymatic activity under acidic conditions. Additionally, our results also showed the rSeCP enzyme activity was inhibited by metal irons in a dose-dependent manner. The results were similar with those reported by Fricker et al. [54]. The physiological concentrations of metal ions are able to modulate the activity of Cathepsin L, even under conditions simulating an extracellular level. It seems that the role of metal ions is important for regulating SeCP enzyme activity.

*S*. *erinaceieuropaei* cysteine protease can hydrolyze Hb, IgG and collagen, and may be associated with digestion of host tissue in invasion, migration and pathogenesis [17,55]. Considering the broad specificity of SeCP against various host proteins (e.g., Ig and Hb), the main biological role of SeCP may be related with digestion of host’s protein for parasite’s nutrition [17,47,55]; this is consistent with the role of cysteine proteases during blood meal feeding. In this study, our results indicated that the rSeCP could hydrolyze Hb, IgG and fibronectin. Combined with the SeCP expressed only in penetrating and migratory stages in the host tissues [44], SeCP might have a potential role in tissue invasion and/or migration. It has been shown that cleaving host IgG is a mechanism of escaping host immune responses utilized by helminthes [55,56]. The cleaving of IgG and fibronectin by rSeCP suggests a possible role for the SeCP in helping plerocercoids evade immune activity by modulating host immune response. The neutralization of SeCP enzymatic activity might have harmful effects on the parasite. To determine whether neutralizing rSeCP activity will interfere with the function and protection of SeCP, the neutralization test and *in vitro* ADCC assay were performed in this study. Our results showed that the enzymatic activity of rSeCP can be neutralized by anti-rSeCP antibodies, suggesting that anti-rSeCP antibodies participated in the killing of infective plerocercoids. Although the immunization of mice with rSeCP induced a Th2-predominant immune responses (high levels of IgG1) and anti-rSeCP antibodies have the potential capability to kill plerocercoids in ADCC reactions, the plerocercoids can survive for a long time in infected mice. However, the SeCP excreted or secreted by plerocercoids during natural infection produced only partial protective immunity. Like most parasites (e.g., *Taenia solium*, *Wuchereria bancrofti* and *Trichinella spiralis*), during the long parasitism and evolution, the plerocercoids might have developed multiple strategies of immune escape to survive in hosts, such as cleaving host IgG, shedding the surface tegument, and producing neutralizing or blocking antibodies [37,56,57].

To evaluate the sensitivity and specificity of rSeCP for detecting anti-plerocercoid antibodies, rSeCP-ELISA was used for detection of serum samples from patients with sparganosis, and the results were compared with ES-ELISA. The results showed that the sensitivity of rSeCP-ELISA and ES-ELISA for detecting anti-plerocercoid antibodies was 100% (20/20), but the specificity of rSeCP-ELISA was significantly greater than that of ES-ELISA (*P* <0.01). The results demonstrated that rSeCP might be a potential antigen for serodiagnosis of sparganosis.

In conclusion, the present study demonstrated that SeCP is a plerocercoid stage-specific protein and located in the teguments and parenchymal tissues of plerocercoids. rSeCP has cysteine protease enzymatic activity, and functions to degrading various host proteins. Immunization with rSeCP induced a Th2-predominant immune responses and anti-rSeCP antibodies had the potential to kill plerocercoids in an ADCC assay. rSeCP had a high sensitivity and specificity for detecting anti-plerocercoid antibodies, and might be a potential antigen for serodiagnosis of sparganosis.

## Supporting Information

S1 Table Results of antibody-dependent cellular cytotoxicity (ADCC) assay against sparganum at 48 h after incubation. (DOC)
