# Supplementary material for: Characterization of Spirometra erinaceieuropaei Plerocercoid Cysteine Protease and Potential Application for Serodiagnosis of Sparganosis
Source: PLoS Negl Trop Dis. 2015 Jun 5;9(6):e0003807. doi: 10.1371/journal.pntd.0003807 (PMC4457932; doi:10.1371/journal.pntd.0003807)
Supplement: S1 Table — (DOC) [file pntd.0003807.s001.doc]

**S1 Table 1** Results of antibody-dependent cellular cytotoxicity (ADCC) assay against spargamum at 48h after incubation

| **Groups (pooled sera from 5 animals)** | **Live** | **Dead** | **Total** | **% Cytotoxicity**  **(Mean± SD)** |
| --- | --- | --- | --- | --- |
| spargana + anti-rSeCP sera + MPM | 4 | 6 | 10 |  |
|  | 3 | 7 | 10 |  |
|  | 2 | 8 | 10 |  |
|  |  |  |  | 70.00±10.00 |
| spargana + sparganum- mouse infected sera + MPM | 3 | 7 | 10 |  |
|  | 3 | 7 | 10 |  |
|  | 1 | 9 | 10 |  |
|  |  |  |  | 76.67±11.55* |
| spargana + normal mouse sera + MPM | 7 | 3 | 10 |  |
|  | 8 | 2 | 10 |  |
|  | 8 | 3 | 10 |  |
|  |  |  |  | 23.33±5.77** |
| spargana + PBS + MPM | 8 | 2 | 10 |  |
|  | 9 | 1 | 10 |  |
|  | 9 | 1 | 10 |  |
|  |  |  |  | 13.33±5.77*** |

In these assays, spargana were incubated with pooled anti-rSeCP sera and 1×10^5^ mouse peritoneal macrophage (MPM).

* Statistically significant ( P＞0.05) compared to mouse infection sera.

**Statistically significant ( P＜0.01) compared to normal mouse sera.

***Statistically significant ( P＜0.01) compared to PBS controls.
